# Emerging Infections Program as Surveillance for Antimicrobial Drug Resistance 

**DOI:** 10.3201/eid2109.150512

**Published:** 2015-09

**Authors:** Scott K. Fridkin, Angela A. Cleveland, Isaac See, Ruth Lynfield

**Affiliations:** Centers for Disease Control and Prevention, Atlanta, Georgia, USA (S.K. Fridkin, A.A. Cleveland, I. See);; Minnesota Department of Public Health, St. Paul, Minnesota, USA (R. Lynfield)

**Keywords:** antimicrobial resistance, health care–associated infection, surveillance, Emerging Infections Program, EIP, bacteria

## Abstract

Across the United States, antimicrobial drug–resistant infections affect a diverse population, and effective interventions require concerted efforts across various public health and clinical programs. Since its onset in 1994, the Centers for Disease Control and Prevention Emerging Infections Program has provided robust and timely data on antimicrobial drug–resistant infections that have been used to inform public health action across a spectrum of partners with regard to many highly visible antimicrobial drug–resistance threats. These data span several activities within the Program, including respiratory bacterial infections, health care–associated infections, and some aspects of foodborne diseases. These data have contributed to estimates of national burden, identified populations at risk, and determined microbiological causes of infection and their outcomes, all of which have been used to inform national policy and guidelines to prevent antimicrobial drug–resistant infections.

## Abstract 2

This program is a national resource for data on the epidemiology of antimicrobial drug–resistant infections and characteristics of isolates.

The 1992 Institute of Medicine report Emerging Infections: Microbial Threats to Health in the United States describes the ability of microbes to adapt, the development of antimicrobial drug resistance, and the importance of recognizing and monitoring emerging microbial threats to human health (*1*). In response, because of the recognized need for more accurate surveillance to detect and address emerging microbial health threats, in 1994 the Centers for Disease Control and Prevention (CDC) Emerging Infections Program (EIP) was established as a collaboration of CDC and state health departments and academic partners. EIP works collaboratively across different programs and disease areas at CDC to deliver critical data that the program is well suited to obtain (*2*).

## EIP as an Antimicrobial Drug Resistance Surveillance System

EIP is grounded in performing active population-based and laboratory-based surveillance. EIP staff regularly query laboratories serving the populations under surveillance (i.e., they perform active case finding) to ensure the reporting of all cases of the selected diseases occurring in the residents of the population under surveillance. EIP investigators then abstract clinical and demographic data from medical records of many patients. To minimize underreporting and ensure complete case ascertainment, they also audit laboratories. For many of the diseases, isolate characterization, including typing and antimicrobial drug susceptibility testing, is done at a central laboratory. Although it is resource intensive, EIP antimicrobial drug resistance surveillance has 4 key attributes: flexibility to adapt to new antimicrobial drug resistance threats, design that enables estimates of the burden of disease (representing large diverse metropolitan areas), collection and delineation of resistant strains, and the ability to follow trends over time. In addition, EIP provides a platform for studies to determine risk factors for antimicrobial drug–resistant disease or to evaluate the effectiveness of public health interventions aimed at preventing antimicrobial drug–resistant infections. Because these data from the EIP have greatly advanced the public health knowledge base of a wide spectrum of antimicrobial drug–resistant infections, the EIP is considered a key antimicrobial drug resistance surveillance platform. For example, EIP contributed data that allowed for national estimates of 10 of the 18 urgent, serious, and concerning pathogens highlighted in the CDC report Antibiotic Resistance Threats in the United States, 2013 (*3*).

## Examples of Antimicrobial Drug Resistance Surveillance and Research in EIP

The Active Bacterial Core surveillance system (ABCs) was one of the initial core areas of the EIP. ABCs tracks invasive (defined as occurring in a sterile site) bacterial infections. During the 1990s in the United States, concern about *Streptococcus pneumoniae* resistance to penicillin increased. From the beginning of ABCs, *S. pneumoniae* isolates were tested by broth microdilution and serotyped at 1 of 3 reference laboratories (CDC, University of Texas Health Science Center, or Minnesota Department of Health). In 2000, nonsusceptibility of *S. pneumoniae* to penicillin peaked (http://www.cdc.gov/abcs/reports-findings/survreports/spneu00.html), coincident with the introduction of the 7-valent pneumococcal conjugate vaccine (PCV7) for routine use in young children. Mathematical modeling with ABCs data predicted that, by 2004, in the absence of an intervention, 41% of invasive pneumococcal isolates would be dually nonsusceptible to penicillin and erythromycin (*4*). Notably, in 1998, of the penicillin- nonsusceptible isolates, 78% were serotypes included in PCV7, and because the vaccine eliminated nasopharyngeal colonization with vaccine serotypes, invasive disease caused by vaccine serotypes declined not only among vaccinated children but also among persons in other age groups (*5**,**6*). However, after widespread use of PCV7, serotype 19A (absent from PCV7 vaccine) became more prominent and more frequently resistant, resulting in increased resistant invasive disease; these results were shared in real time with the Advisory Council for Immunization Practices to help inform the vaccine industry about relevant changes in serotypes. In 2010, the 13-valent pneumococcal conjugate vaccine, which included this serotype, was licensed, and resistance once again declined, as measured by EIP (http://www.cdc.gov/abcs/reports-findings/survreports/spneu13.pdf).

Other community invasive bacterial infections evaluated by EIP include those caused by group A *Streptococcus*, group B *Streptococcus*, and *Neisseria meningitidis.* Program highlights have included demonstration of a plasmid carrying the *erm*T methylase gene that conferred macrolide and inducible clindamycin resistance in group A *Streptococcus* (*7*); demonstration of macrolide and inducible clindamycin resistance in group B *Streptococcus*, leading to changes in recommendations for intrapartum antimicrobial drug prophylaxis for penicillin-allergic women, by CDC and professional organizations (*8*); and the finding of ciprofloxacin-resistant *N. meningitidis* in Minnesota and North Dakota (*9*), prompting a local change in prophylaxis recommendations.

In a similar fashion, through routine collection and evaluation of isolates, the EIP Healthcare Associated Infections–Community Interface activity (*10*) has produced critical knowledge for informing approaches to clinical management of candidemia. Data collected from Georgia and Maryland EIP sites during 2008–2011 showed that, during a period of general adoption of fluconazole prophylaxis in infants of extremely low birthweight to prevent neonatal candidemia, rates of candidemia in infants markedly declined (*11*) and levels of fluconazole resistance among *Candida* spp. bloodstream isolates remained relatively stable (*12*). However, subsequent analyses identified increases in echinocandin-resistant and multidrug-resistant *Candida* infections during 2008–2012 (*13*); evaluation of the emergence of echinocandin resistance in *C. glabrata* and development of molecular testing to detect resistance could be accomplished because of the systematic collection of such isolates in EIP (*14*).

As carbapenem-resistant *Enterobacteriaceae* (CRE) emerged rapidly in the United States and elsewhere, there was no clear method or mechanism in place for hospitals or health departments to develop an accurate assessment of CRE in their area. Susceptibility definitions were evolving, laboratory methods differed, and different resistance mechanisms had been associated with carbapenem resistance. In 2010, as part of the Healthcare Associated Infections-Community Interface portfolio of EIP projects (*10*), the Georgia and Minnesota EIP sites piloted methods for CRE surveillance. A review of epidemiologically defined CRE-case isolates characterized at CDC for carbapenemase genes enabled analysis of different case definitions to maximize specificity or sensitivity to most likely predict the presence of a carbapenemase gene. This information is helping to inform a national case definition for CRE (*15*) to be used by state health departments and hospital infection control staff for reporting and responding to CRE infections.

Several attributes of EIP are clear in the success of the methicillin-resistant *Staphylococcus aureus* (MRSA) surveillance activity. First, in the late 1990s, after the deaths of 4 children in Minnesota and North Dakota who did not have traditional health care–associated risk factors for MRSA were reported (*16*), EIP demonstrated flexibility by modifying operations to expand case ascertainment to include nonsterile sites (in addition to the more typical approach of sterile sites) and to characterize the epidemiology of community-associated MRSA through work at 4 EIP sites. In 2001, the Georgia, Maryland, and Minnesota EIP sites (*17*) reported that infections were more likely among young children and black persons and that only 6% of infections were invasive (compared with 77% reported as skin and soft tissue infections). Notably, almost three quarters of community-associated MRSA infections were treated with antimicrobial drugs to which the strains were resistant. Second, EIP contributed to a more standardized surveillance approach by providing definitions for case types: community-associated (no health care risk factors), health care–associated community-onset (within 3 days of hospital admission), and hospital-onset (*18*). Most (58%) invasive disease was health care–associated community-onset, 27% was hospital-onset, and only 14% was community-associated. Third, population-based surveillance enabled extrapolation to the US population. In 2005, invasive MRSA was estimated to have caused 94,000 infections and 18,600 deaths, a number that was >2-fold higher than cases and deaths caused by invasive pneumococcal disease. Fourth, EIP comprehensive case finding and ability to categorize characteristics of patients brought underappreciated populations at risk to attention; the largest burden of disease requiring the next wave of prevention activity is among patients recently discharged from the hospital (*19*).

In several ways, the EIP system provides a platform for work on antimicrobial drug resistance among infections transmitted commonly by food. CDC conducts the human side of the National Antimicrobial Resistance Monitoring System for Enteric Bacteria (NARMS) (*20*) in collaboration with the Food and Drug Administration and the US Department of Agriculture.

FoodNet sites also participate in NARMS surveillance for antimicrobial resistance in *Campylobacter* spp. and, along with health departments in all other states, in *Salmonella* spp., *Shigella* spp., and *Vibrio* spp. (*21*). The FoodNet sites also collaborate with the Food and Drug Administration retail meat sampling for NARMS. Its purpose is to monitor the prevalence of selected bacteria, including *Salmonella* and *Campylobacter* spp., in meat and poultry and to track resistance in these bacteria. FoodNet has also collaborated with NARMS on studies of human illnesses. FoodNet conducted studies of *Campylobacter* infections, which showed that eating poultry was a risk factor for quinolone-resistant infections and that diarrhea persisted longer in patients with these resistant infections; this finding contributed to the withdrawal of approval for use of fluoroquinolones in poultry (*22**,**23*). All of these data have helped inform ongoing approaches taken by the Department of Health and Human Services to eliminate the use of antimicrobial drugs for growth promotion in food animals and to bring all therapeutic uses under veterinary oversight (*24*).

## Conclusions

As an antimicrobial drug resistance surveillance system, EIP is unique because it takes advantage of a design to enable much more useful analyses and public health assessments than simply defining the proportion of clinical isolates processed by a laboratory that are resistant to a specified antimicrobial drug. Data from EIP provide the clinical and epidemiologic context needed to quantify and compare clinically relevant infections and relative burden of disease with other public health priorities. Because EIP surveillance is population based, robust national estimates can be made, and these have been proven very useful for informing national policy. Also, the systematic collection and study of isolates have informed surveillance definitions and methods for routine public health activities, as well as direction for industry to develop pharmacologic and nonpharmacologic interventions. The infrastructure provides the flexibility needed to respond to new resistance problems by having a committed and experienced collaboration among federal, state, and local public health institutions with clinical laboratories and academic institutions.
